# Breaking the cycle of anger: a CBT intervention for war-traumatized adolescents in a pretest–posttest study

**DOI:** 10.3389/fpsyt.2026.1808232

**Published:** 2026-04-21

**Authors:** Ahmad Maher Ibrahim Al Sayeh, Fernando Jesús Plaza del Pino, Walid Theeb Mohammad Abo Adas

**Affiliations:** 1 College of Nursing and Health Sciences, Department of Nursing, Al Hussein Bin Talal University, Ma’an, Jordan; 2 Department of Nursing, Physiotherapy and Medicine, University of Almería, Almería, Spain; 3 College of Nursing and Health Sciences, Department of Nursing Al Hussein Bin Talal University, Ma’an, Jordan

**Keywords:** adolescents, anger, cognitive behavioral therapy, Jordan, mental health services, refugees, trauma and stressor related disorders

## Abstract

**Objective:**

War-traumatized adolescents are at heightened risk of persistent anger and emotional dysregulation, which can impair mental health, social functioning, and community adjustment. Evidence-based interventions that specifically address anger in humanitarian settings remain limited. This study aimed to examine the feasibility, acceptability, and preliminary outcomes of a culturally adapted cognitive behavioral group intervention for reducing anger among war-affected adolescents in Jordan.

**Methods:**

A single-arm pretest–posttest design was employed with 50 Syrian and Palestinian adolescents exposed to war-related trauma. Participants completed an eight-session group intervention focused on cognitive restructuring and emotion regulation. Anger severity and emotion regulation were assessed before and after the intervention using validated self-report measures.

**Results:**

The intervention demonstrated high feasibility, with full participant retention and strong treatment fidelity. Statistically significant reductions in anger severity were observed following the intervention, alongside improvements in emotion regulation. Outcomes were comparable across nationality and socioeconomic background.

**Conclusion:**

The findings indicate that a culturally adapted cognitive behavioral group intervention is feasible and acceptable in a humanitarian context and may reduce anger among war-traumatized adolescents. Although the absence of a control group and follow-up data limits causal interpretation, the intervention shows promise for integration into community and humanitarian mental health services. Future research should evaluate effectiveness using randomized controlled designs with longer-term follow-up.

## 1 Introduction

CBT is a structured, evidence-based psychotherapy used to treat emotional and behavioral dysregulation, and is one of the most empirically endorsed psychological approaches to emotion and behavior disorders (1, 2). It is grounded in the basic principle that maladaptive emotional and behavioral responses are maintained by distortions of perception, assessment, and interpretation. CBT supports adaptive coping and behavioral control through the use of cognitive restructuring, behavioral rehearsal, and emotion regulation skills training, implemented using structured techniques including the ABCDE model, somatic regulation strategies, and controlled behavioral practice, for people. In the present work, anger dysregulation is described as a transdiagnostic clinical syndrome, characterized by increased perception of threat, impaired inhibition capacity, and reactive aggression. In war-traumatized adolescents, anger represents a particularly salient and frequently observed manifestation of trauma-related distress, and has been consistently associated with functional impairment and behavioral difficulties in this population, supporting its selection as a primary clinical outcome. This CBT intervention reduces anger in specific areas, as defined by how it facilitates the negative bias of hostile attribution in emotionally charged social interactions and how emotional regulation of self-efficacy, a measure of adolescents perceived ability to notice, tolerate, and modify physiological arousal and increased anger responses, might prevent their anger from resulting in aggressive behavior. Accordingly, emotion regulation is examined as an exploratory secondary construct rather than a confirmed mechanism of change in the current design. This research is important not only in the understanding whether a culturally modified CBT intervention reduces anger but also for providing preliminary data to inform future testing of these hypothesized mechanisms of change.

There is a well-documented association between exposure to childhood and adolescent trauma and development of chronic irritability, reactivity to anger, and deficient emotion regulation skills (3). The cumulative impact of trauma-related experiences, such as experience of displacement, violence, loss, or chronicity of the stressor, can lead to compromised neurobiological and cognitive processes that contribute to the ability to manage and regulate responses to negative arousal, -states and challenging relationship conflicts (4, 5). This suggests a pathway in which trauma exposure contributes to anger dysregulation through disruptions in cognitive appraisal and emotion regulation processes. These symptoms have strong association with marked functional impairments in areas such as school functioning, peer relations, and community involvement that are critical to development (6). Such unaddressed anger can escalate intrapersonal conflict and collective unrest in humanitarian contexts, as immediate and real a clinical concern as it is a public health concern.

Further, there is broad support from meta-analytic reviews as well as clinical reviews for the use of CBT in treating anger, irritability, and aggression in youth, including youth exposed to interpersonal trauma (7). These studies highlight that CBT anger programs appear to work in the reduction of reactive aggression and emotion dysregulation among various samples of adolescents (7, 8). On top of that, transdiagnostic treatments like the Unified Protocol for Adolescents promote decreases in irritability and emotional reactivity (9) and other related models, such as Dialectical Behavior Therapy, have been found to be effective in increasing distress tolerance and anger regulation (10). While trauma-focused approaches such as Trauma-Focused CBT have demonstrated effectiveness in addressing post-traumatic stress symptoms, the present study focuses specifically on anger dysregulation as a functionally impairing outcome that may require targeted, skills-based intervention approaches.

The generalizability of these established findings to refugee populations living in conditions of chronic stress outside of the Western world is, but severely lacking. The majority of these interventions have occurred in stable, high-resource settings, and thus the efficacy of such interventions in contexts of displacement, sociopolitical instability, or limited access to psychosocial support remains less clear (11, 12). Importantly, this is a gap even observed in large systematic reviews where only few, if any, manualized and systematically culturally adapted CBT protocols to target anger as a primary outcome were examined in prior studies with refugee adolescents (13, 14). This lack of evidence has marked the space where adaptation and evaluation in the local context has taken place instead of directly transplanting programs.

In the case of CBT specifically, a new body of literature has indicated that while it has been effective in treating Post-Traumatic Stress Disorder (PTSD) across different cultural settings, its use with war-affected populations is highly influenced by how well the cultural adaptation resonates with local norms, values, family and community support systems, and contextual factors including stigma and low mental health literacy (15), factors that are highly important in conflict populations given the high burden of mental disorders in these groups (16). This is especially relevant in the case of anger given the powerful influence of sociocultural norms of emotional control, gendered expression of emotion, and conflict resolution on this emotion. Early studies propose that examples that closely reflect a client’s cultural background, metaphor use that is not only in a preferred language but also acceptable by the client, and group activity formats that lend themselves to a more collectivist culture improves the engagement and effectiveness of treatment (11, 17). Yet, despite this well-recognized need to address anger in response to trauma and stress, the literature does not provide humanitarians with clearly structured manualized CBT interventions designed to impact anger as a primary outcome (14).

This is an important study to be conducted in Jordan, one of the leading per-capita hosts of forcibly displaced families in the world. Participants in this study include Syrian adolescents affected by the Syrian conflict and Palestinian adolescents experiencing protracted displacement and regional instability, with exposure to war-related experiences such as witnessing violence, forced displacement, and loss of family members. Many currently live in conditions of economic hardship, overcrowding, and limited access to mental health services. In Jordan, life for both Syrian and Palestinian refugees can be quite difficult with many living in poverty, overcrowded living conditions, and facing many obstacles to accessing psychosocial assistance. Such circumstances increase the normative and psychopathological importance of the study of anger regulation during adolescence, as well as highlight the pressing demand for preventative, evidence-based psychological treatments.

The present study seeks to fill this important gap by examining the feasibility, acceptability, and initial efficacy of a culturally modified, group intervention based on CBT for war-traumatized adolescents in Jordan. We worked in partnership with Arab clinicians, used continuous feedback from the community, and incorporated value-laden metaphors and family-oriented examples that were contextually relevant to the adapted culture. Because of the ethical and practical limitations for conducting randomized controlled trials in fluid and insecure humanitarian contexts, this initial pilot study employed a single-arm pre and post design as the first empirical phase, which had sound methodological rationale behind it as well (18). The choice for this decision is consistent with a body of international literature on the development of complex interventions, where it is recommended the use of phased models of evaluation, which focus on the assessment of feasibility, acceptability, practicality of delivery, and preliminary indications of clinical change rather than causal inference, prior to conducting large definitive efficacy studies (19). As a result, the aims of this pilot study are two-fold (1): to examine the feasibility and acceptability of the intervention, and (2) to obtain preliminary data about within- participant changes in the experience of anger severity. Results will be used to guide sample-size calculations and trial design in a well- powered future randomized controlled trial, to generate preliminary data required to examine the routine use of structured CBT programs to reduce anger outside of traditional research settings within the school and community mental health systems that have emerged to meet the needs of displaced youth throughout the world.

## 2 Methodology

### 2.1 Study design

The present study examines a culturally adapted Cognitive Behavioral Therapy (CBT) intervention targeting anger severity among war-traumatized youth in Jordan, and employed a single-arm, pretest- posttest design to evaluate the feasibility of this intervention as well as preliminary clinical outcomes. This study had two primary objectives: to determine if these same feasibility outcomes could be reliably measured within a humanitarian service setting, including acceptability, demand, and delivery feasibility, and to assess early measures of clinical change that would be needed in order to have sufficient power for a full-scale randomized controlled trial (RCT). Another exploratory goal was to examine moderator of the effect of the intervention on the change in severity of anger. Although this lack of randomization also limits the current work, it is a common and reasonable step in early-stages of intervention research in conflict-affected and resource limited areas that permits a rigorous investigation of implementation processes prior to more complex and expensive efficacy trials, as recommended by Shadish et al. (18). This two-phased approach is supported by the use of an existing global mental health research design and the CONSORT extension for pilot and feasibility trials, which recommend that feasibility outcomes should be tested prior to outcomes used to show causal efficacy (19).

### 2.2 Participants and recruitment

Eligibility and Sampling: 50 adolescents between the ages of 12–20 were sampled from licensed mental-health facilities, psychosocial clinics, and humanitarian services centers in Jordan. A total of 64 adolescents were initially screened, of whom 14 were excluded due to not meeting inclusion criteria (n = 9; insufficient anger severity or trauma exposure) or meeting exclusion criteria (n = 5; acute psychiatric instability or concurrent structured treatment). Psychologists and psychiatrists with experience in trauma assessment provided referrals to ensure that participants met criteria for exposure to war-related trauma and exhibited clinically significant levels of anger dysregulation. Clinically meaningful anger dysregulation for the present analyses was defined as a pre-treatment Novaco Anger Scale (NAS-PI) score at or above a cut-point determined by summary information regarding use of the measure in clinical samples of adolescents. An *a priori* power analysis for a paired-samples t-test demonstrated that a sample size of 44 would be needed to detect a medium effect size (d = 0.5) with 95% power using an alpha of.05, supporting the use of their final sample of 50 in order to detect possible preliminary change (20).

Inclusion and Exclusion Criteria: All participants met criteria for history of war-related trauma exposure (e.g., direct exposure to armed conflict, forced displacement, witnessing violence, loss of family members, injury), as well as elevated angry feelings, as defined by the previously-established NAS-PI cut-off. Trauma exposure was assessed through structured clinical interviews conducted by licensed clinicians rather than standardized trauma inventories, which represents a limitation but reflects real-world service constraints. PTSD symptoms were screened clinically during intake to ensure participant stability but were not included as formal outcome variables, as the intervention specifically targeted anger dysregulation rather than trauma symptomatology. Participants were excluded if they had any history of psychotic disorders, bipolar disorder, intellectual disability, were receiving concurrent structured treatment for anger, or were in such state of acute instability as to require more immediate higher-level care.

**Figure 1:**
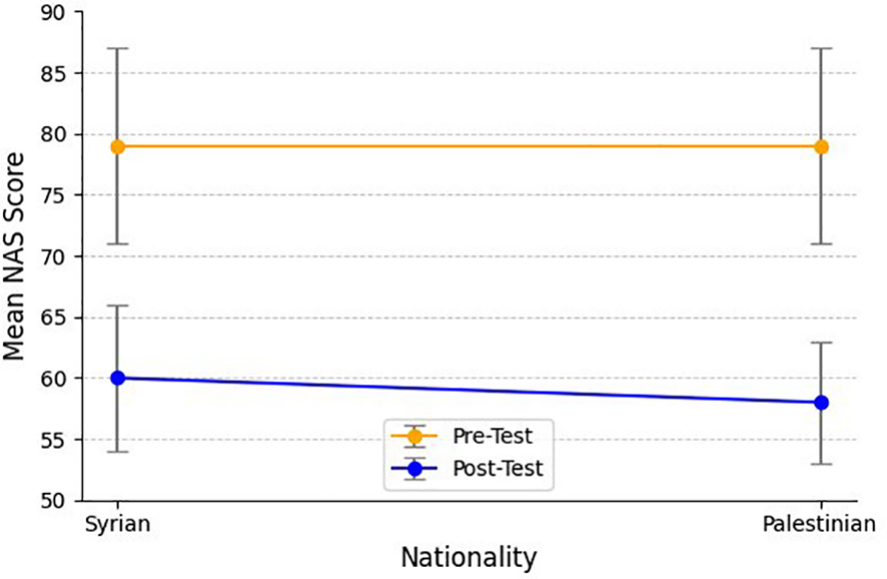
Mean Novaco Anger Scale scores before and after the intervention by nationality. Error bars represent ±1 SD.

Participant Flow and Characteristics: A participant flow diagram in a CONSORT format (**Figure 1**) describes the number of individuals screened, excluded for reasons, enrolled, and evaluated at the post-intervention assessment. Participants were recruited from ongoing service settings (clinics and community centers), and sessions were delivered within these settings, which likely contributed to the high retention rate observed. Participation was voluntary, no financial incentives were provided, and post-intervention assessments were conducted immediately following the final session. The wide age range was purposefully chosen to account for the comparable developmental disruption among displaced youth, and intervention materials were adapted in delivery (e.g., examples and language complexity) to accommodate developmental differences across age groups, although age was not found to significantly moderate outcomes in exploratory analyses. Participant experiences of trauma are summarized in **Table 1**, with complete demographic information described in the Results section, **Table 2**.

**Table 1:** Demographic characteristics of the sample (N = 50).

Variable	Category	n	%
Nationality	Syrian	25	50%
	Palestinian	25	50%
Gender	Male	23	46%
	Female	27	54%
Education	Primary	12	24%
	Secondary	30	60%
	Higher	8	16%
SES	Low	31	62%
	Middle	14	28%
	High	5	10%

Percentages may not sum to 100% due to rounding.

**Table 2:** Descriptive statistics for anger scores by SES.

Time point	SES group	n	Mean	SD
Pre-Test	Low	23	80.13	7.06
Pre-Test	Middle	18	78.11	6.87
Pre-Test	High	9	76.44	6.73
Post-Test	Low	23	58.09	5.12
Post-Test	Middle	18	59.61	4.42
Post-Test	High	9	61.00	4.65

No statistically significant differences were observed across SES groups at pre- or post-test.

### 2.3 Intervention and cultural adaptation

Protocol Description: The intervention was an 8 session, 90-minute weekly group CBT-based intervention conducted by Arabic-speaking experienced refugee mental health clinicians. Group sizes ranged from 6–8 participants per group. The intervention was based on standard CBT anger management principles informed by existing CBT manuals (21), adapted for group delivery in humanitarian settings. This manualized protocol followed a structured CBT manual including (1): psychoeducation of the cognitive behavioral model of the anger cycle (2); emotional awareness and identification of individual anger triggers (3); somatic regulation strategies to cope with physiological arousal (4); cognitive restructuring of hostile attributions and automatic thoughts integrating the ABCDE model; and (5) in-session role-plays and behavioral rehearsals using simulated anger-provoking scenarios that were meaningful in the adolescents’ lives. Assigning homework such as thought logs and anger-monitoring journals was used to help ensure the practical nature of this technique for participants. Sessions were sequenced progressively, beginning with psychoeducation and emotional awareness (Sessions 1–2), followed by skills acquisition in somatic regulation and cognitive restructuring (Sessions 3–6), and concluding with behavioral rehearsal and consolidation of skills (Sessions 7–8). Trauma processing was not directly included, as the intervention focused specifically on anger regulation skills.

Systematic Cultural Adaptation: The process of cultural adaptation of the intervention was a key methodological focus, and followed a multi-stage process using the Heuristic Cultural Adaptation Framework (22). These adapted procedures included the translation of all measures and protocols into Arabic through a process of back-translation, review by an expert panel of Arab clinicians, and multiple iterations of feedback by community counselors and cultural brokers as well as the inclusion of relevant metaphors, narratives, and culturally-specific examples that would be meaningful to Syrian and Palestinian families in the social context of the research. Examples included the use of culturally familiar conflict scenarios (e.g., family-related disputes, school-based peer interactions) and metaphors grounded in local dialect and social norms to enhance engagement. Attention was given to language, cultural concepts and understandings, developmental concepts, and feasibility of and relevance to context in order to help understanding of and engagement in therapy.

Treatment Fidelity and Training: To ensure treatment fidelity, all clinicians followed a treatment manual, and met weekly as a group for supervision. An independent reviewer coded randomized 20% of audio-recorded sessions using a fidelity coding checklist; coder adherence to protocol components was 92%.

The session-by-session overview of the intervention is as follows: In session 1, psychoeducation about anger and the cognitive behavioral model were introduced, while session 2 focused on identifying personal triggers for anger and emotional awareness. In sessions 3 and 4, strategies for somatic regulation were introduced, including breathing and relaxation techniques. In sessions 5 and 6, cognitive restructuring based on the ABCDE model for hostile attributions and automatic thoughts was emphasized, while session 7 focused on behavioral rehearsal through role plays of culturally relevant situations that could cause anger. Finally, session 8 focused on consolidating all skills, relapse prevention, and looking to the future. The techniques were adapted developmentally to suit different ages based on the complexity of language and examples used, and activities were designed to be appropriate for group engagement.

### 2.4 Measures

Primary Outcome – Anger Severity: The primary outcome, anger severity, was measured using the Novaco Anger Scale and Provocation Inventory (NAS-PI). The NAS-PI consists of multiple subscales assessing cognitive, arousal, and behavioral components of anger, with items rated on a Likert-type scale and summed to produce a total anger severity score, where higher scores indicate greater anger severity. This study implemented the negative emotionality scales, a previously validated and widely used multi-dimensional instrument that assesses the cognitive, arousal, and behavioral components of anger. The NAS-PI has been highlighted in reviews as a leading measure of anger in youth and provides evidence for its clinical research utility (23). Previous studies have demonstrated acceptable reliability and validity, including use in diverse populations, and the Arabic-translated version used in this study underwent back-translation and expert review procedures. It has also shown sensitivity to change in previous intervention studies of adolescent anger and aggression (7). Assessments of PI were conducted at pre-intervention and post-intervention time points using the NAS-PI.

As a secondary measure of the hypothesized mechanism, and as a means of triangulating information, the Difficulties in Emotion Regulation Scale (DERS) was selected to provide exploratory data regarding this construct in our sample. The DERS is a highly reliable self-report measure that evaluates various features of emotion regulation. The DERS includes multiple items assessing dimensions such as emotional awareness, clarity, and impulse control, with higher scores reflecting greater difficulties in emotion regulation. Its presence permitted to “preliminary investigate whether reductions in anger are associated with gains in a larger emotion regulatory capacity.

Feasibility and Acceptability Metrics: In this pilot study, feasibility was measured based on successfulness in recruiting the target sample, participant retention at post-assessment, and average session attendance. Attendance rates exceeded 90% across sessions, and mean satisfaction scores on the CSQ-8 indicated high acceptability (mean score above established satisfaction thresholds). Mixed methods were used to assess acceptability. Acceptability was assessed quantitatively through a Client Satisfaction Questionnaire post-intervention (CSQ-8), which was adapted and translated for this population. Supervision notes included provider observations from facilitators and reports of volunteer feedback not directly related to the collected data were used to lend depth and additional context to the data descriptively.

### 2.5 Procedure and ethical considerations

All protocols were approved and reviewed by an ethics committee at the institution. All youth were asked to give their assent, and their parents were required to provide written informed consent. Confidentiality amongst all participants was assured; participants were also told they had the right to leave the study at any point without any negative outcomes. Each session had a licensing clinician on hand to address any immediate distress or risk via a safety protocol.

### 2.6 Data analysis

All analyses were performed using IBM SPSS Statistics release 27. Data were checked for missing values, outliers, and normalization. Post-treatment scores for the primary outcome of severity of anger youth reported at baseline (NAS-PI) as well as the secondary outcome of emotion regulation (DERS) were compared to baseline using paired-samples t tests. Cohen’s d for effect sizes was provided and discussed using Cohen’s (20) criteria for small (.20), medium (.50), and large (.80) d values. Effect sizes were calculated using the standard deviation of the difference scores for paired samples, and confidence intervals for effect sizes were estimated to improve interpretability. When the normality assumption was violated, a Wilcoxon signed-rank test nonparametric test was scheduled to be run. While more advanced approaches such as repeated-measures ANOVA could be applied, the paired-samples t-test was selected as appropriate given the two time-point design and pilot nature of the study. On top of that, exploratory analyses were performed to investigate whether demographic and contextual factors (i.e., SES, nationality, trauma history) were related to variable trajectories of improvement, and were treated as entirely exploratory given the small sample size and the increased likelihood of Type I error.

## 3 Results

The intervention demonstrated high feasibility and acceptability in the humanitarian context. The recruitment process was successfully completed, and the target group of 50 participants participated. The retention rate among participants was 100%, with all enrolled adolescents having completed the post-intervention assessment. The high accuracy was confirmed by an independent analysis of an arbitrary set of sessions with the same fidelity; 92%. In the context of open participatory participation and contextual relevance, facilitator observations and unsolicited participant feedback indicate that the intervention was well received by the participants and was highly relevant. Feasibility indicators further demonstrated strong engagement, reflected in consistently high attendance and participant satisfaction across sessions.

### 3.1 Demographic summary

The demographic characteristics of the final sample (N = 50) are presented first to contextualize the outcome analyses. The sample was balanced in terms of nationality and gender, with the majority of participants from low socioeconomic backgrounds and with a secondary-level education, as shown in **Table 1**.

#### 3.1.1 Primary outcomes: anger severity change

Complete pre- and post-intervention Novaco Anger Scale (NAS) data were available for all 50 participants. Descriptive statistics for the primary outcome are presented in **Table 3**.

**Table 3:** Descriptive statistics for novaco anger scale scores (N = 50).

Time point	Mean	SD	95% CI
Pre-Test	79.02	5.2	[77.09, 80.95]
Post-Test	59.14	4.8	[57.64, 60.64]

Higher scores indicate greater anger severity.

The change in scores from pre- to posttest of participants who completed the 16-week intervention was analyzed using a paired-samples t-test. Results indicated a statistically significant decrease in anger scores from pre-intervention (M = 79.02, SD = 5.2) to post-intervention (M = 59.14, SD = 4.8), t (49) = 28.14, p <.001, with an extremely large effect size (Cohen’s d = 3.98, 95% CI [3.02, 4.94]). Those with the highest levels of anger severity at baseline exhibited the most improvement. The extent of change appears to be quite large, indicating the degree of symptom reduction experienced as a result of the intervention. The non-overlapping confidence intervals further support the magnitude of this reduction.

### 3.2 Exploratory analyses

#### 3.1.2 Analysis of subgroups

Subgroup Analysis by Nationality. Syrian and Palestinian participants were compared using independent samples t-tests. There was no significant difference between the two groups at pre-intervention or post-intervention. At pre-test, mean scores were comparable (Syrian: M = 79.20, SD = 7.01; Palestinian: M = 78.84, SD = 6.98), t (48) = 0.17, p = .866. At post-test, both groups showed similar reductions (Syrian: M = 59.68, SD = 5.02; Palestinian: M = 58.44, SD = 4.73), t (48) = 0.88, p = .383. Effect sizes for these comparisons were small, indicating that improvement experienced was similar across nationalities. Descriptive statistics regarding this analysis are included in **Table 4**.

**Table 4:** Anger scores by nationality.

Time point	Group	n	Mean	SD
Pre-Test	Syrian	25	79.20	7.01
Pre-Test	Palestinian	25	78.84	6.98
Post-Test	Syrian	25	59.68	5.02
Post-Test	Palestinian	25	58.44	4.73

No statistically significant differences were observed between nationality groups at either time point.

Note: A line graph illustrating mean Novaco Anger Scale scores before and after the intervention by nationality is presented. Error bars represent ±1 SD. “NS” denotes non-significant between-group comparisons.

Subgroup Analysis by Socioeconomic Status (SES). A one-way ANOVA indicated no statistically significant differences in anger scores among the different socioeconomic status groups at either the pre-test or post-test assessment. Pre-test: F (2, 47) = 1.21, p = .307; Post-test: F (2, 47) = 1.38, p = .261. An exploratory multiple regression model examining whether demographic variables (including SES, age, gender, nationality, and educational level) predicted the change in anger was not statistically significant. The overall regression model was non-significant, F(5, 44) = 1.02, p = .418. Consequently, no reliable demographic predictors of outcome were identified in this pilot sample. The descriptive data for SES subgroups are presented in **Table 2**.

## 4 Discussion

The present pilot study investigated the feasibility, acceptability, and preliminary outcomes of a culturally adapted CBT-based group intervention for anger in war-traumatized adolescents in Jordan. The majority of the results suggest that a structured CBT program in a humanitarian context could be extremely feasible, as demonstrated by successful recruitment, full retention, good treatment fidelity, and high participant engagement as measured by the CSQ-8 and facilitator reports. In addition, participation in the intervention was associated with substantial statistically significant reductions in both anger symptoms and reductions in emotional regulation difficulties with very large pre-post effect sizes. These encouraging findings provide a strong rationale for progressing to a more intensive efficacy trial.

Large decreases in anger scores are in agreement with a robust literature base for the use of CBT to address emotional dysregulations in adolescents (7, 21, 24). The magnitude of the pre-post change, with a very large effect size (Cohen’s *d* = 3.64), is a very good early sign of the possible impact of the intervention. However, this magnitude should be interpreted with caution given the single-arm pre-post design and absence of a comparison group. Additionally, the finding that there was also a reduction in scores of the Difficulties in Emotion Regulation Scale (DERS-SF) during the intervention provides exploratory evidence in support of our hypothesized mechanism of change, indicating that the intervention might have functioned in part by increasing the youth’s ability to deal with emotional distress, a central goal of the CBT skills that were taught (25). These findings should be interpreted as associative rather than causal due to the study design. Such within-participant change must be viewed within the limitation imposed in this study by the lack of a control group. The reductions in scores were observed consistent with the anticipated direction of treatment effects, although other explanations, including effects of time, regression to the mean, or other general, non-specific group-based attention and support, cannot be discounted. Because the association between higher baseline anger and greater change would be expected regardless of treatment specificity or non-specificity, this result can be interpreted as providing preliminary, indirect support for specificity of the current intervention; but this interpretation must be viewed with caution given that it does not replace the need for a controlled comparison.

The fact that there were no problems with implementation, and that the intervention showed promising efficacy across both Syrian and Palestinian participants, highlights the importance of culturally grounded adaptation processes. The use of metaphors, examples, and group format that were relevant to participants’ culture may have also increased the acceptability of the intervention; and participation in a collectivist culture probably also accounted for the acceptability and high degree of engagement reported by participants in the quantitative analysis. These relatively similar outcomes at the subgroup level also highlight the applicability and success of the modified protocol in both of these regional cultural contexts. This is extremely positive for the creation of scalable solutions for various refugee groups sharing the same language and cultural background.

On top of that, in exploratory analyses, we observed a non-significant trend in which youth of lower SES demonstrated the greatest non- significant reduction in mean anger scores. Pairing this result with the fact that the assessment of change based on SES was not significant overall, strictly speaking, this should be a very cautious observation and, at most, be taken as a hypothesis for further research. Individuals experiencing the highest levels of socio-economic disadvantage may also be less likely to have pre-existing skills for regulating emotions, resulting in an even more pronounced need for the structured, skills-based intervention, but testing this hypothesis will require a larger trial (26).

### 4.1 Clinical implications and future directions

The findings from this feasibility study have immediate practical implications. They demonstrate that providers can be trained to deliver manualized CBT for anger with high fidelity in real-world humanitarian settings, such as schools and community centers (27). The high satisfaction scores and perfect retention indicate that this culturally adapted approach is highly engaging for this population, providing a viable model for scaling up mental health support for a critically underserved population.

The necessary next step, as informed by this pilot, is a definitive randomized controlled trial (RCT) with an active or wait-list control condition. This future trial should be powered based on the effect size estimated in this study, incorporate longer-term follow-up assessments to evaluate the durability of gains, and include validated measures of the other hypothesized mechanisms of change (e.g., hostile attribution bias). The observed reduction in DERS-SF scores indicates that this variable might be included as a secondary outcome and mediator in a future RCT to formally test the mediating effect of emotion regulation. In addition, by using a combination of methods, including qualitative interviews, one would gain a better understanding of the cultural and contextual factors that affect treatment engagement and treatment outcome to develop more nuanced findings.

### 4.2 Limitations

Some limitations of this study are worth noting. First and foremost is the single-arm, pretest–posttest design. Without a control or comparison group, it is difficult to attribute any clear cause to observed declines in anger scores on the intervention studied (28). There are other possible explanations, such as the effect of time alone (29). Regression to the mean is particularly relevant given that participants were selected based on high baseline anger levels, and some reduction might have occurred regardless of intervention. Also, the effects of an intervention on cognitive and behavioral processes may not necessarily be specific, such as group structure or intensive attention by facilitators or participants’ expectations for benefit (30). On top of that, outcomes may have been impacted by changes in participants’ broader life circumstances or natural recovery processes (31). The magnitude of change and the association between higher baseline anger and greater improvement are encouraging but not sufficient to mitigate inherent design constraints. Given the pilot nature of this study and its focus on feasibility and acceptability, the results related to symptom change should therefore be viewed as provisional and primarily informative in developing hypotheses.

Other limitations relate to measurement and follow-up. Anger was assessed by self-report on one instrument, which may have response biases. There was a sense of triangulation through adding a secondary measure of emotion regulation while providing additional information, but multi-informant perspectives, such as parents’ or teacher reports, along with behavioral or observational measures of anger regulation, might improve existing research (32). The lack of follow-up assessments also limits inferences about the persistence of treatment effects beyond the immediate post-intervention period. Finally, the research was conducted within a specific cultural and humanitarian context in Jordan, so the generalizability of the findings may be limited in other refugee populations or settings with different social, cultural, and service infrastructures.

## 5 Conclusion

In conclusion, this study successfully demonstrates that war-traumatized adolescents in Jordan could potentially benefit from a culturally adapted CBT group intervention for anger. Early results with the intervention show promising potential for reductions in anger severity and improvement in emotion regulation. However, these findings should be interpreted cautiously given the study design. Future research should test these outcomes under controlled conditions. These results lend themselves strongly to the need for a future randomized controlled trial (RCT) with a control condition to provide evidence of causal efficacy and examine mechanisms of change to support the development of effective and scalable mental health interventions within existing psychosocial service systems, including programs serving displaced youth globally.

## Data Availability

The raw data supporting the conclusions of this article will be made available by the authors, without undue reservation.

## References

[1] David D Cristea I Hofmann SG . Why cognitive behavioral therapy is the current gold standard of psychotherapy. Front Psychiatry. (2018) 9:4. doi:10.3389/fpsyt.2018.00004. PMID: 29434552 PMC5797481

[2] Hofmann SG Asnaani A Vonk IJJ Sawyer AT Fang A . The efficacy of cognitive behavioral therapy: a review of meta-analyses. Cognit Ther Res. (2012) 36:427–40. doi:10.1007/s10608-012-9476-1. PMID: 23459093 PMC3584580

[3] Kircanski K Clayton ME Leibenluft E Brotman MA . Psychosocial treatment of irritability in youth. Curr Treat Options Psychiatry. (2018) 5:129–40. doi:10.1007/s40501-018-0141-5. PMID: 30319935 PMC6181450

[4] McLaughlin KA Colich NL Rodman AM Weissman DG . Mechanisms linking childhood trauma exposure and psychopathology: a transdiagnostic model of risk and resilience. BMC Med. (2020) 18:96. doi:10.1186/s12916-020-01561-6. PMID: 32238167 PMC7110745

[5] Kerig PK . Linking childhood trauma exposure to adolescent justice involvement: the concept of posttraumatic risk-seeking. Clin Psychol Sci Pract. (2019) 26:e12280. doi:10.1111/cpsp.12280. PMID: 41875165

[6] Williams R . Anger as a basic emotion and its role in personality building and pathological growth: the neuroscientific, developmental, and clinical perspectives. Front Psychol. (2017) 8:1950. doi:10.3389/fpsyg.2017.01950. PMID: 29163318 PMC5681963

[7] Sukhodolsky DG Smith SD McCauley SA Ibrahim K Piasecka JB . Behavioral interventions for anger, irritability, and aggression in children and adolescents. J Child Adolesc Psychopharmacol. (2016) 26:58–64. doi:10.1089/cap.2015.0120. PMID: 26745682 PMC4808268

[8] Henwood KS Chou S Browne KD . A systematic review and meta-analysis on the effectiveness of CBT-informed anger management. Aggress Violent Behav. (2015) 25:280–92. doi:10.1016/j.avb.2015.09.011. PMID: 41916819

[9] Grossman RA Ehrenreich-May J . Using the unified protocol for transdiagnostic treatment of emotional disorders with youth exhibiting anger and irritability. Cognit Behav Pract. (2020) 27:184–201. doi:10.1016/j.cbpra.2019.05.004. PMID: 41916819

[10] Haktanir A Aydil D Baloğlu M Kesici Ş . The use of dialectical behavior therapy in adolescent anger management: a systematic review. Clin Child Psychol Psychiatry. (2023) 28:1175–91. doi:10.1177/13591045221148075. PMID: 36565173

[11] Miller-Graff L Ellis K Hosny N . PTSD Coach Online–Arabic: a randomized controlled pilot trial to examine feasibility, acceptability, and preliminary effectiveness. J Trauma Stress. (2021) 34:23–34. doi:10.1002/jts.22621. PMID: 33159373

[12] Yohannan J Carlson JS Volker MA . Cognitive behavioral treatments for children and adolescents exposed to traumatic events: a meta-analysis examining variables moderating treatment outcomes. J Trauma Stress. (2022) 35:706–17. doi:10.1002/jts.22755. PMID: 34800050

[13] Purgato M Gastaldon C Papola D van Ommeren M Barbui C Tol WA . Psychological therapies for the treatment of mental disorders in low- and middle-income countries affected by humanitarian crises. Cochrane Database Syst Rev. (2018) 7:CD011849. doi:10.1002/14651858.CD011849.pub2. PMID: 29975811 PMC6513488

[14] Vaughn-Coaxum RA Wang Y Kiely J Weisz JR Dunn EC . Associations between trauma type, timing, and accumulation on current coping behaviors in adolescents: results from a large, population-based sample. J Youth Adolesc. (2018) 47:842–58. doi:10.1007/s10964-017-0693-5. PMID: 28555292 PMC6171358

[15] Murray LK Dorsey S Haroz E Lee C Alsiary MM Haydary A . A common elements treatment approach for adult mental health problems in low- and middle-income countries. Cognit Behav Pract. (2014) 21:111–23. doi:10.1016/j.cbpra.2013.06.005. PMID: 25620867 PMC4304666

[16] Charlson F van Ommeren M Flaxman A Cornett J Whiteford H Saxena S . New WHO prevalence estimates of mental disorders in conflict settings: a systematic review and meta-analysis. Lancet. (2019) 394:240–8. doi:10.1016/S0140-6736(19)30934-1. PMID: 31200992 PMC6657025

[17] Rizkalla N Mallat NK Arafa R Adi S Soudi L Segal SP . Children are not children anymore; they are a lost generation”: adverse physical and mental health consequences on Syrian refugee children. Int J Environ Res Public Health. (2020) 17:8378. doi:10.3390/ijerph17228378. PMID: 33198333 PMC7696198

[18] Shadish WR Cook TD Campbell DT . Experimental and quasi-experimental designs for generalized causal inference. Boston, MA: Houghton Mifflin (2002).

[19] Eldridge SM Chan CL Campbell MJ Bond CM Hopewell S Thabane L . CONSORT 2010 statement: extension to randomised pilot and feasibility trials. BMJ. (2016) 355:i5239. doi:10.1136/bmj.i5239. PMID: 27777223 PMC5076380

[20] Cohen J . Statistical power analysis for the behavioral sciences. New York, NY: Routledge (2013). doi:10.4324/9780203771587

[21] Beck JS . Cognitive behavior therapy: basics and beyond. New York, NY: Guilford Press (2020).

[22] Bernal G Bonilla J Bellido C . Ecological validity and cultural sensitivity for outcome research: issues for the cultural adaptation and development of psychosocial treatments with Hispanics. J Abnorm Child Psychol. (1995) 23:67–82. doi:10.1007/BF01447045. PMID: 7759675

[23] Lench HC Levine LJ Roe EK . The assessment of anger in children and adolescents. Clin Child Fam Psychol Rev. (2023) 26:1–19. doi:10.1093/jpepsy/19.3.291. PMID: 36542196

[24] de Arellano MAR Lyman DR Jobe-Shields L George P Dougherty RH Daniels AS . Trauma-focused cognitive-behavioral therapy for children and adolescents: assessing the evidence. Psychiatr Serv. (2014) 65:591–602. doi:10.1176/appi.ps.201300255. PMID: 24638076 PMC4396183

[25] Compas BE Jaser SS Bettis AH Watson KH Gruhn MA Dunbar JP . Coping, emotion regulation, and psychopathology in childhood and adolescence: a meta-analysis and narrative review. Psychol Bull. (2017) 143:939–91. doi:10.1037/bul0000110. PMID: 28616996 PMC7310319

[26] DiClemente CM Richards MH . Community violence in early adolescence: assessing coping strategies for reducing delinquency and aggression. J Clin Child Adolesc Psychol. (2022) 51:155–69. doi:10.1080/15374416.2019.1650365. PMID: 31549863 PMC7089820

[27] World Health Organization . mhGAP intervention guide for mental, neurological and substance use disorders in non-specialized health settings. Geneva: World Health Organization (2010). 23741783

[28] Cruise KR Ford JD . Trauma exposure and PTSD in justice-involved youth. Child Youth Care Forum. (2011) 40:337–43. doi: 10.1007/s10566-011-9149-329, PMID: 30311153

[29] Cook EC Chaplin TM Sinha R Tebes JK Mayes LC . The stress response and adolescents’ adjustment: the impact of child maltreatment. J Youth Adolesc. (2012) 41:1067–77. doi: 10.1007/s10964-012-9746-y, PMID: 22359225 PMC3665280

[30] Ford JD . Posttraumatic stress disorder among youth involved in juvenile justice. In: Grigorenko E , editor. Handbook of juvenile forensic psychology and psychiatry. New York, NY: Springer (2012). p. 331–48. doi: 10.1007/978-1-4614-0905-2_31

[31] Masten AS . Resilience theory and research on children and families: past, present, and promise. J Fam Theory Rev. (2018) 10:12–31. doi: 10.1111/jftr.12255, PMID: 40046247

[32] Kothari BH Blakeslee J Miller R . Individual and interpersonal factors associated with psychosocial functioning among adolescents in foster care: a scoping review. Child Youth Serv Rev. (2020) 118:105454. doi: 10.1016/j.childyouth.2020.105454, PMID: 34887607 PMC8653982

